# Bioactive Compounds and Antifungal Activity of Leaves and Fruits Methanolic Extracts of *Ziziphus spina-christi* L.

**DOI:** 10.3390/plants11060746

**Published:** 2022-03-11

**Authors:** Amany A. El-Shahir, Deiaa A. El-Wakil, Arafat Abdel Hamed Abdel Latef, Nora H. Youssef

**Affiliations:** 1 Department of Botany and Microbiology, Faculty of Science, South Valley University, Qena 83523, Egypt; nora_hassan@sci.svu.edu.eg; 2 Biology Department, Facutly of Science, Jazan University, Jazan 82817, Saudi Arabia; de107@yahoo.com; 3 Plant Pathology Research Institute, Agricultural Research Center, Giza 12619, Egypt

**Keywords:** *Ziziphus*, *Alternaria*, tomato fruits, antifungal activity, methanolic extracts

## Abstract

*Zizyphus spina-christi* L. has antimicrobial properties because of the presence of biologically active compounds. *Alternaria* is an opportunistic pathogen that causes leaf spots, rots, and blights on a variety of plant parts. This study aimed to reduce the usage of synthetically derived fungicides. Identification of the bioactive components present in leaves and fruits methanolic extracts of *Z. spina-christi* was performed using high-performance liquid chromatography (HPLC) and gas chromatography-mass spectrometry (GC-MS). The efficacy of the two methanol extracts was tested against (a) in vitro fungal growth and (b) pathogenicity control on non-wounded and wounded tomato fruits. The results revealed that gallic acid and ellagic acid were the major components in leaves extract while quercetin was the major component in fruits extract. In addition, Phenol, 2,5-bis(1,1-dimethylethyl) (40.24%) and Decane, 2-methyl-(18.53%) were the most abundant components in the leaf extract, and the presence of D-mannonic acid, 2,3,5,6-tetrakis-o-(trimethylsilyl), and γ-lactone (22.72%) were major components in fruits extract. The methanolic extracts of *Z. spina-christi* leaves and fruits demonstrated significant antifungal activity against the growth of *Alternaria alternata*, *A. citri*, and *A. radicina* with variable inhibition percentages at different concentrations. Pathogenicity was increased when the skin was injured, as expected. Both extracts reduced the percentage of infected fruits.

## 1. Introduction

Plant pathogenic fungi are responsible for 20–40% of all known plant diseases. Fungicides are the most common way to prevent a fungal infection from causing excessive yield or quality loss. Chemical fungicides are unquestionably effective in controlling fungal infections, but they will face major limitations in the future due to several significant concerns. Furthermore, chemical fungicides have the potential to cause deadly medical consequences, such as cancer [1,2].

The *Rhamnaceae* family includes the *Ziziphus* genus. There are 100 deciduous and evergreen trees and shrubs in this genus, which can be found in tropical and subtropical climates all over the world [3].

*Zizyphus spina-christi* L. leaves and fruits extracts had antimicrobial or antibacterial potential against *Staphylococcus aureus*, *Candida albicans*, *Bacillus subtilis*, and *Escherichia coli* [4,5]. However, to the best of our knowledge and an extensive literature review search, the antifungal efficacy of the methanol extract of the leaves and fruits of *Z. spina-christi* on *Alternaria* sp. has not yet been investigated extensively, although reports have shown that *Z. spina-christi* was potentially a good source of antimicrobial compounds and other functional groups. The *Ziziphus* genus has previously been found to include a variety of functional substances including vitamin C, amino acids, triterpene acids, polysaccharides, polyphenols, flavonoids, triterpene acids, saponins, alkaloids, indole derivatives, and fatty acids [6]. The use of these compounds especially for applications in crop protection was not as common as in the medical field [7].

Generally, methanol extract is more active than other extracts because it has good extraction efficiency for various bioactive compounds [8]. Moreover, Adamu et al. [9] and Roy et al. [10] reported that the methanolic extract of *Z. spina-christi* roots and fruits revealed antifungal activity against dermatophytes, including *Trichophyton rubrum*, *T. mentagaphytes*, *Microsporum canis*, *Aspergillus fumigatus*, and *Candida albicans*.

*Alternaria* sp. causes early blights, which are serious destroyers that inflict serious damage to produce. In nearly 400 distinct host species, it has been linked to the spread of leaf spots and other diseases. *Alternaria* sp. could infect many crops in the field and throughout the postharvest period, resulting in losses due to fruit and vegetable rot. The production of *Alternaria* mycotoxins was recorded in many naturally infected fruits and vegetables, such as tomatoes, apples, grapes, blueberries, oranges, lemons, mandarins, and olives. It is an opportunistic pathogen that attacks several plant parts, causing leaf spots, rots, and blights [11]. Because of their high moisture content, high nutrient content, and thin peel, tomato fruits (*Lycopersicon esculentum*) are particularly sensitive to *Alternaria* sp. infection, which results in postharvest losses [12]. Plant extracts and natural products are gaining popularity since they do not represent a health risk or pollute the environment. These items are also less expensive than chemicals and have few, if any, detrimental side effects on the hosts. Chemical fungicides have several problems, including high acute and chronic toxicity, extended breakdown durations, accumulation in the food chain, and an extension of their ability to kill both beneficial and destructive pests. Plant extracts minimize these disadvantages [13]. As a result, the current focus is shifting to the biological control of plant diseases and biological control programs to acquire critical information on the application’s long-term impacts [14,15,16].

This study aimed to reduce the usage of synthetically derived fungicides by determining the antifungal activities of the leaves and fruits methanol extract of *Z. spina- christi* against *Alternaria alternata*, *A. citri*, and *A. radicina*. The efficacy of methanol extracts of this plant was tested against (a) in vitro fungal growth and (b) pathogenicity control on non-wounded and wounded tomato fruits. High-performance liquid chromatography (HPLC) and gas chromatography-mass spectrometry (GC-MS) techniques were used to identify the phytochemical components in two extracts.

## 2. Materials and Methods

### 2.1. Chemicals

Standard grade agar was purchased from Meron, Cochin-682005, India. Chloramphenicol and dextrose were obtained from Bio Vision, South Milpitas Blvd, Milpitas, CA, USA. Solvents of HPLC grade were used. Gallic acid, chlorogenic acid, catechin, methyl gallate, coffeic acid, syringic acid, pyrocatechol, rutin, ellagic acid, coumaric acid, vanillin, ferulic acid, naringenin, quercetin, cinnamic acid, kaempferol, and hesperetin were obtained from (Sigma-Aldrich, Merck, Darmstadt, Germany). Mancozeb was purchased from Misr for Agricultural Development Company, Sadat City, Monufia Governorate, Egypt.

### 2.2. Collection of Plant Samples

Fresh healthy samples of leaves and fruits of *the Ziziphus spina-christi* L. plant were collected from the campus of South Valley University, Qena, Egypt (26°11′32″ N, 32°44′43″ E). Samples were collected, and then they were immediately transferred to the laboratory for washing with distilled water. Each of the leaves or fruits samples were left to air-dry at 25 °C under sterilized conditions for three weeks. The dried leaves and fruits were ground separately to a powder form and stored in a tight glass container for further investigations.

### 2.3. Preparation of Plant Extracts

A total of 80 g of the dried leaves and fruits powders of (*Z. spina-christi* L.) were separately extracted by methanol (100%) by soaking in 500 mL of the solvent, held with occasional shaking, and left overnight. The mixture was filtrated using Whatman filter paper No.1, and the methanolic extracts were dried by leaving the filtrate at room temperature at 25 °C for 6 days in a safety cabinet, where they were completely dried [17]. The crude methanolic extracts of the two plant parts were used for antifungal assays and identification of bioactive compounds (Figure S1A,B). The estimation of the particle size was not one of the objectives of this study.

### 2.4. High-Performance Liquid Chromatography (HPLC) Analysis

HPLC analysis was carried out for the quantitative identification of polyphenols compounds in two crude methanolic extracts of *Z. spina-christi* L by (an Agilent 1260 series Santa Clara, CA, USA), which was equipped with DAD (G1315D 1260 DAD VL), MWD (G1365D 1260 MWD VL) and FLD (G1321C 1260 FLD), and consisted of an autosampler (G1329B 1260 ALS) with a pump (G1311C 1260 Quat pump VL) and a thermostat (G1330B 1290). The separation was performed by Eclipse C18 column (4.6 mm × 250 mm i.d., 5 μm) G1316A1260 TCC. The mobile phase was composed of water (A) and 0.05% trifluoroacetic acid in acetonitrile (B) at a flow rate of 1 mL/min. The mobile phase was programmed consecutively in a linear gradient as follows: 0 min (82% A); 0–5 min (80% A); 5–8 min (60% A); 8–12 min (60% A); 12–15 min (82% A); 15–16 min (82% A); and 16–20 (82%A). The multi-wavelength detector was monitored at 280 nm. The injection volume was 5 μL for each of the sample solutions. The standard solutions were prepared for the 17 investigated phenolic compounds: gallic acid, chlorogenic acid, catechin, methyl gallate, coffeic acid, syringic acid, pyrocatechol, rutin, ellagic acid, coumaric acid, vanillin, ferulic acid, naringenin, quercetin, cinnamic acid, kaempferol, and hesperetin. A stock standard solution (500 mg/L) of each phenolic compound was prepared in methanol by weighing out approximately 0.025 g of the analyte into a 50 mL volumetric flask and diluting to the respective volume. The mixed standard solution was prepared by diluting the mixed stock standard solutions in methanol to give a concentration of 50 mg/L for each polyphenol. All standard solutions were stored in the dark at 5 °C and were stable for at least three months. The column temperature was maintained at 40 °C.

### 2.5. Gas Chromatography-Mass Spectrometry Analysis (GC-MS)

The dried methanolic extracts were resuspended in 50 µL of N, O-Bis (trimethylsilyl) trifluoroacetamide (BSTFA) incubated in a Dry Block Heater at 70 °C for 30 min.

The phytochemical components in methanol leaves and fruits extract of *Z. spina-christi* were determined by GC-MS analysis. The GC-MS system used (Agilent Technologies, Santa Clara, CA, USA) was equipped with a gas chromatograph (7890B, G3440B), a mass spectrometer detector (5977A, MSD G7040A), and headspace (7697 A headspace sampler G4556_64000). The GC was prepared with an HP-5MS column (30 m × 0.25 mm internal diameter and 0.25 μm film thickness). Analyses were conceded with helium as the carrier gas at a flow rate of 1.0 mL/min at a splitless injection volume of 1 µL. The next temperature program was as follows: 50 °C for 1 min, increasing at 10 °C/min to 300 °C, and apprehended for 20 min. The injector and detector were caught at 250 °C. Mass spectra were acquired by electron ionization (EI) at 70 eV by a spectral range of m/z 30–700 and a solvent gradient ACN: H_2_O: trifluoroacetic acid (TFA). The solvent delay was 9 min. The mass temperature was 230 °C and Quad 150 °C. The spectrum fragmentation pattern was compared to those recorded in Wiley and NIST Mass Spectral Library data to identify various constituents.

### 2.6. Fungal Strains Collection and Identification

Three *Alteranria* species were employed in antifungal activity investigations (plant pathogenic fungi): *A. alternata* MW850355, *A. citri* MW851893, and *A. radicina* MW852019. These fungi were isolated from infected tomato fruits with black spot symptoms. Baiting procedure according to [18] was employed for the isolation of fungi from black spot symptomatic tomato fruits.

### 2.7. In Vitro Antifungal Activity

On potato dextrose agar (PDA)-filled Petri dishes, fungal spores were cultivated and incubated for 7 days at 28 °C. To make the concentration of (C2 50 mg/mL, C3 100 mg/mL, C4 150 mg/mL, and C5 200 mg/mL), one milliliter of each of the examined extracts was added aseptically and individually to sterile melted PDA media. Plates without extracts (C) and plates with the fungicide Mancozeb 0.3% (C1) were made. For each, a triplicate was performed. The fungal inoculums were put on the agar surface after the plates had cooled. At 28 °C, all Petri plates were incubated for 10 days. Radial growth of fungal mycelium was measured after incubation. Every two days, the colony’s diameter was measured. For growth inhibition, the antifungal activities were assessed as the mean colony diameter (cm) (Figure S2). The inhibition percentages were calculated for each fungal strain when the growth of mycelia in the control plate reached the edge of the Petri plate, after 10 days of incubation. The following formula was used to calculate the percentage of inhibition:

Inhibition% = R − r/R × 100

where (R) represents the radial growth of fungal mycelia on the control plate, and (r) represents the radial growth of fungal mycelia on the plate treated with the leaves and fruits methanolic extract of (*Z. spina-christi* L.).

### 2.8. Determination of Minimal Inhibitory Concentration (MIC)

The minimal inhibitory concentration (MIC) for *Z. spina-christi* L. leaves and fruits extracts essential for maximum inhibition of mycelial growth for *A. alternata*, *A. citri*, and *A. radicina* strains were recorded after 10 days of incubation at 28 °C.

### 2.9. Fungal Pathogenicity and Aggressiveness on Tomato Fruit

The pathogenicity and aggressiveness of *A. alternata*, *A. citri*, and *A. radicina* strains were studied in vivo to see if *Z. spina-christi* L. methanolic extracts had an inhibitory action [19] with modifications. In the experiment, we used healthy tomato fruits (species) of the same size and form. After rinsing the fruits under running water, they were submerged for 2 minutes in a 1% sodium hypochlorite solution. They were rinsed and dried in a laminar flow using sterile distilled water. Each tomato fruit was marked and divided into two zones. A sterile scalpel was used to wound one of the sections (2 mm deep and 3 mm wide), while the other was left uninjured. Each 200 g of fruit was sprayed with 10 mL of 200 mg/mL methanolic extracts of *Z. spina-christi* L. leaves and fruits and then dried in a laminar flow cabinet for 30 min. To achieve uniform distribution, the sprayer was held 30 cm away from the fruit, producing a thin mist. To inoculate each tomato, a 2 mL spore suspension of 7-day-old PDA culture media of *A. alternata*, *A. citri*, and *A. radicina* was employed, both in the wounded and healthy parts. For each treatment, 10 replicates were created. Fruits infected separately with the three strains served as positive controls. Uninoculated fruits sprayed with methanol served as negative controls. To rule out the possibility of methanol having an inhibitory effect, infected fruits were sprayed with methanol alone to generate a third type of control (solvent control). At 25 °C, the fruits were incubated for 5 days. After the incubation time, the number of infected fruits was recorded, and the extent of the lesion was assessed. Pathogenicity was measured by measuring the size of the lesion and aggressiveness by assessing the percentage of infected fruits (Figure S1C).

### 2.10. Statistical Analysis

Data were statistically analyzed using the SPSS program. Analysis of variance (ANOVA) was carried out using a general one-way model.

## 3. Results

### 3.1. Identification of Polyphenols Compounds by HPLC

**Figure 1:**
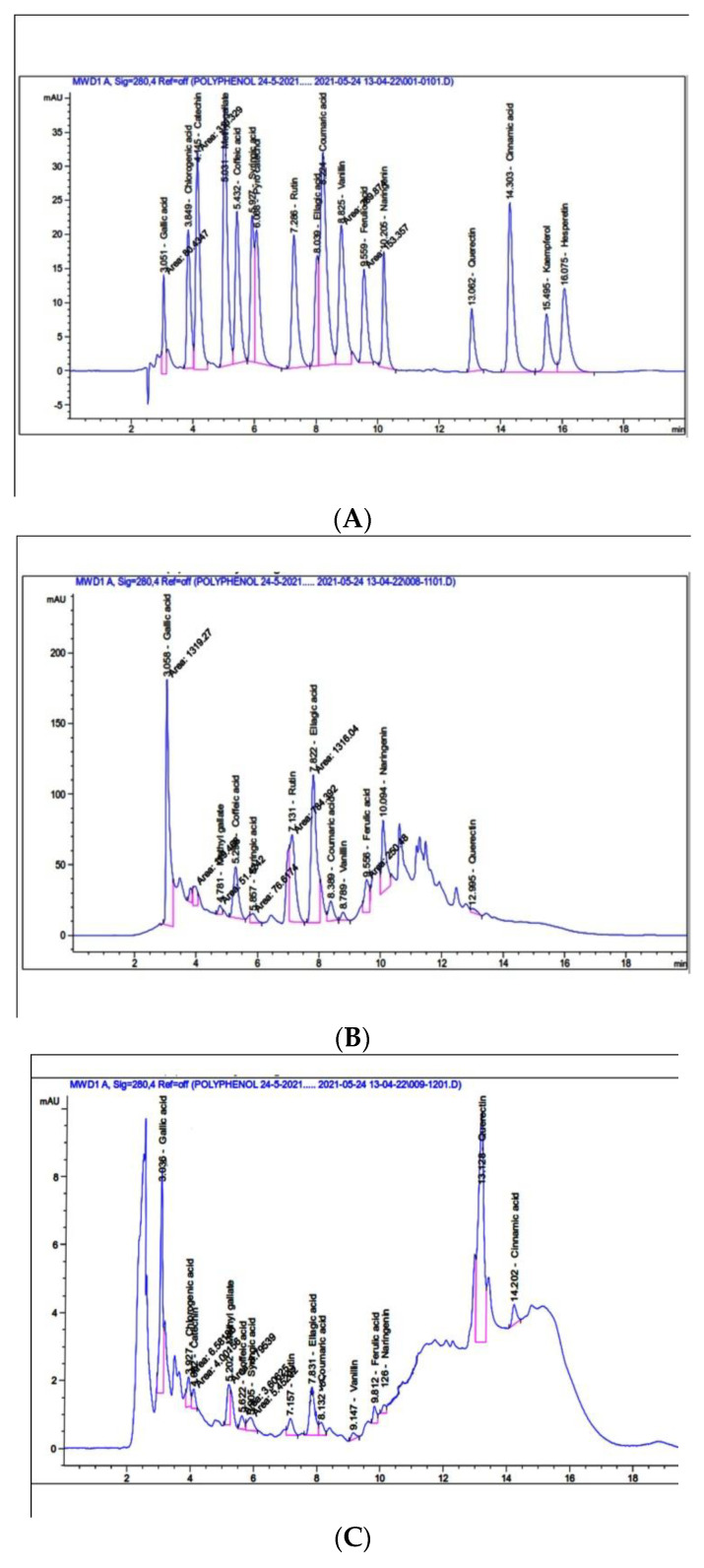
(**A**). HPLC Chromatogram of phenolic compounds reference material (standards), (**B**). HPLC Chromatogram of phenolic compounds in methanolic leaves extract of *Z. spina-christi*, (**C**). HPLC Chromatogram of phenolic compounds in methanolic fruits extract of *Z. spina-christi*.

Seventeen polyphenolic compounds were available in the reference sample, namely: gallic acid, chlorogenic acid, catechin, methyl gallate, coffeic acid, syringic acid, pyrocatechol, rutin, ellagic acid, coumaric acid, vanillin, ferulic acid, naringenin, quercetin, cinnamic acid, kaempferol, and hesperetin (Figure 1A, Table S1). These standard samples were used to identify the polyphenols in the crude leaves and fruits extracts. The results showed that 13 and 14 compounds with different retention times were recognized in the HPLC chromatogram of methanolic extract of leaves and fruits, respectively. The results in Table 1 and Figure 1B,C indicate the phenolic compounds which were detected in methanolic leaves and fruits extracts of *Z. spina-christi* with different concentration levels. The polyphenolic compounds of methanolic extract of leaves contained gallic acid, chlorogenic acid, catechin, methyl gallate, coffeic acid, syringic acid, rutin, ellagic acid, coumaric acid, vanillin, ferulic acid, naringenin, and quercetin with different concentrations compared with standard compounds. Gallic acid and ellagic acid were the predominantly identified components, followed by rutin, then the other polyphenolic compounds. However, the methanolic extract of fruits contained gallic acid, chlorogenic acid, catechin, methyl gallate, coffeic acid, syringic acid, rutin, ellagic acid, coumaric acid, vanillin, ferulic acid, naringenin, quercetin, and cinnamic acid. Quercetin was the main component of polyphenols in *Z. spina-christi* methanolic fruits extract followed by gallic acid. Pyro catechol, kaempferol, and hesperetin were not detected in both crude extracts, while cinnamic acid was absent in the methanolic extract of leaves but present in the methanolic extract of fruits. The phenolic compounds detected in the leaves extract, such as gallic acid, coffeic acid, rutin, ellagic acid, ferulic acid, and naringenin, recorded a marked accumulation versus the standards material.

### 3.2. Phytochemical Analysis by GC-MS Spectroscopy

**Figure 2:**
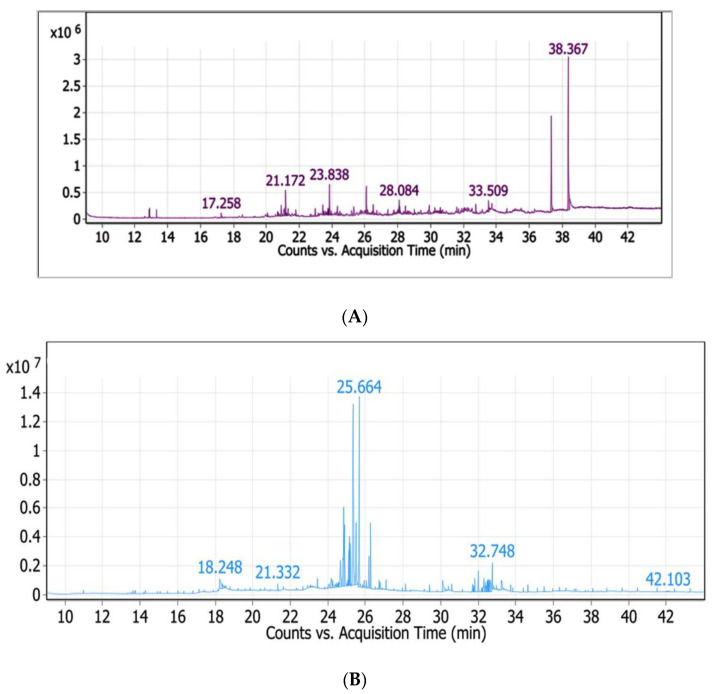
(**A**). GC-MS spectral chromatogram of *Z. spina-christi* leaves extract; (**B**). GC-MS spectral chromatogram of *Z. spina-christi* fruits extract.

The phytochemical components found in the methanol of the leaves and fruits extract of *Z. spina-christi* identified by GC-MS analysis are obtained in Table 2 and Table 3 and Figure 2A,B. The bioactive components with their retention time, molecular formula, and peak area percentage are shown in Table 2 and Table 3. The data of GC-MS spectroscopy of methanol leaves extract revealed the presence of 13 components (Table 2) and the presence of 28 components for methanol fruits extract (Table 3). The GC-MS analysis results have shown the presence of Phenol, 2,5-bis(1,1-dimethylethyl), which is the most abundant component in the leaves extract (40.24%), followed by Decane, 2-methyl- (18.53%), Disulfide, di-tert-dodecyl (14.61%), Nonadecane (7.6%), Aspidosperdin-17-ol,1-acetyl-16-methoxy- (3.1%), 1-Dodecene (2.87%), and Di-t-butyl-4-methylene-2,5-cyclohexadiene-1-one (2.21%). In addition, the most predominant bioactive compounds in the fruits extract is D-mannonic acid, 2,3,5,6-tetrakis-o-(trimethylsilyl)-,.gamma.-lactone, which represents (22.72%); D-Erythro-Pentofuranose, 2-deoxy-1,3,5-tris-O-(trimethylsilyl) (15%); Methyl. alpha-D-glucofuranoside, 4TMSderivative (11.18%); 2,2-Dimethyl-5-[2-(2-trimethylsilylethoxymethoxy)-propyl]-[1,3]dioxolane-4-carbo (8.46%); D-(+)-Turanose, octakis (trimethylsilyl) ether (6.52%); Glucopyranose, 1,2,3,4,6-pentakis-O-(trimethylsilyl)-D- (3.62%); D-Erythrotetrofuranose, tris-O-(trimethylsilyl)- (2.48%); Heptanedioic acid, bis(trimethylsilyl) ester (2.35%); Furan-2-carboxylic acid, 3-methyl-, trimethylsilyl ester (2.27%); and a small quantity of other fatty acids and carbohydrates.

### 3.3. Antifungal Activity

**Figure 3:**
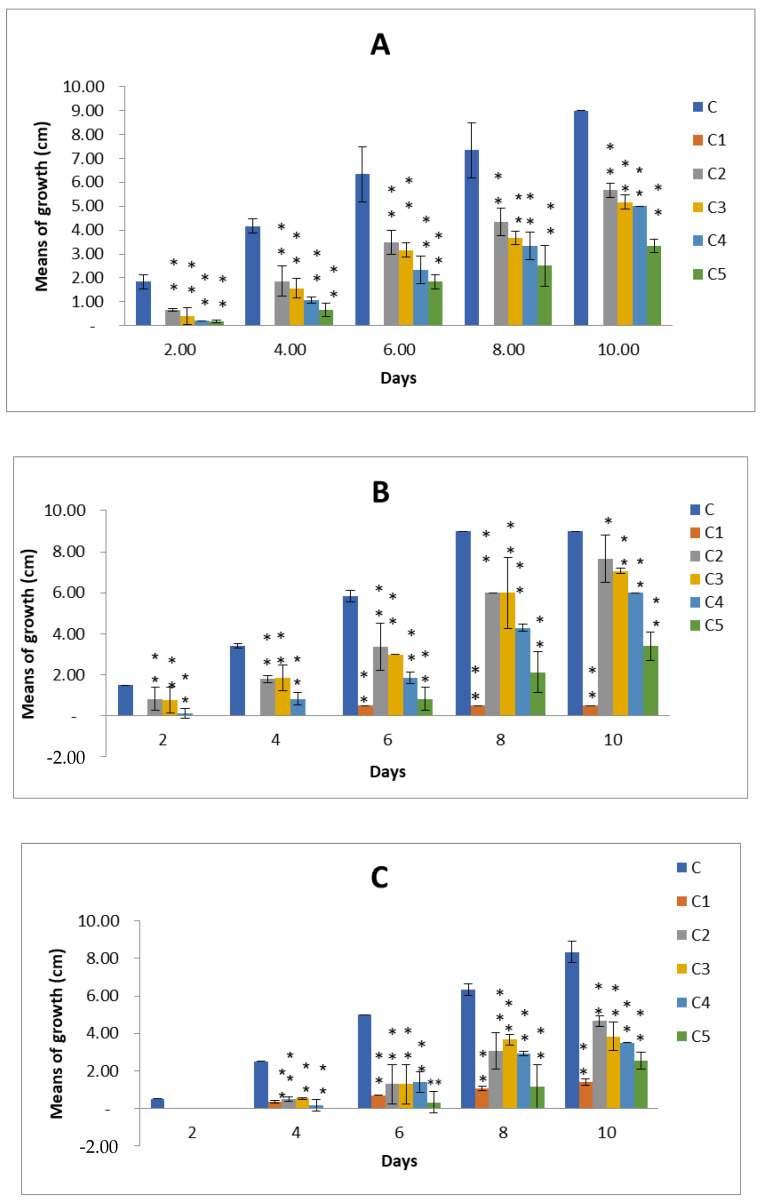
Antifungal activity of leaves extracts of *Zizyphus spina-christi* L. against (**A**) *A. alternata*, (**B**) *A. citri*, and (**C**) *A. radicina* at different concentrations: C is control, C1 Mancozeb, C2 50 mg/mL, C3 100 mg/mL, C4 150 mg/mL, and C5 200 mg/mL. Values are means of three replicates ± standard deviation. Statistical significance of differences compared to control: * Significant at *p* > 0.05, ** Significant at *p* < 0.01 according to Duncan test.

**Figure 4:**
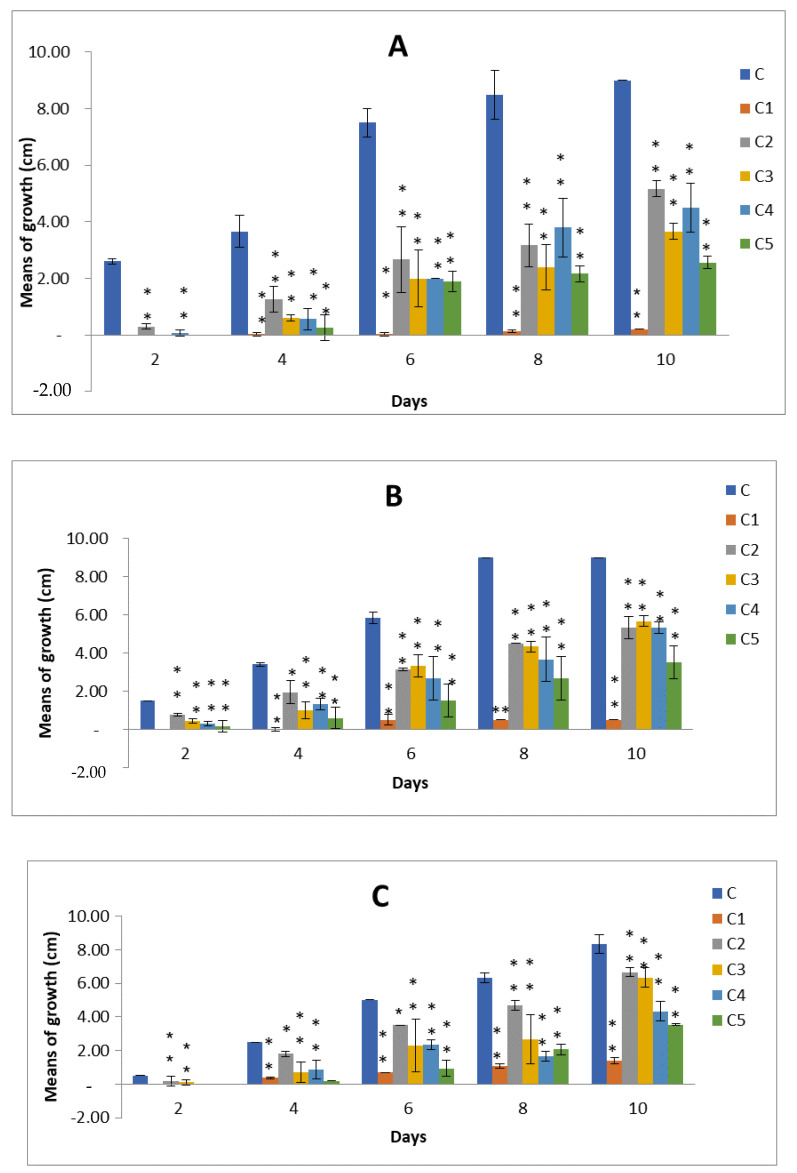
Antifungal activity of fruits extract of *Zizyphus spina-christi* L. against (**A**) *A. alternate*, (**B**) *A. citri,* and (**C**) *A. radicina* at different concentrations: C is control, C1 Mancozeb, C2 50 mg/mL, C3 100 mg/mL, C4 150 mg/mL, and C5 200 mg/mL. Values are means of three replicates ± standard deviation. Statistical significance of differences compared to control: * Significant at *p* > 0.05, ** Significant at *p* < 0.01 according to Duncan test.

Using a growth inhibition assay technique, the *Z. spina-christi* L. leaves and fruits methanolic extracts demonstrated varying antifungal activity against *A. alternata*, *A. citri*, and *A. radicina* at different concentrations. All effects were significant (*p* < 0.05) according to ANOVA. For a total of 10 days of incubation, the antifungal activities were measured as the mean colony diameter (cm) for growth inhibition (every 48 h), and the inhibition percentages were calculated. It was clear from (Figure 3 and Figure 4) that the fungus without any extracts treatment (control) had the biggest colony diameter, which was increased by increasing the incubation period; after 10 days, it was between 8.0 and 9cm. With all of the studied fungi, Mancozeb (0.3%) had the smallest colony diameter, ranging from 0 to 1.40 cm. During the 10-day incubation period, the leaves methanolic extract showed significant antifungal activity at (C2 50mg/mL, C3 100 mg/mL, C4 150mg/mL, and C5 200 mg/mL). After 10 days of incubation, the colony diameters of *A. alternata*, *A. citri*, and *A. radicina* were 3.3, 3.4, and 2.5cm, respectively, with the greatest antifungal activity detected at (C5 200 mg/mL) (Figure 3 and Figure S2). During the 10-day incubation period, the fruits’ extract showed significant antifungal activity against the three tested strains at (C5 200 mg/mL). After 10 days of incubation, C5 200 mg/mL showed significant antifungal efficacy against *A. alternata*, *A. citri*, and *A. radicina* with colony diameters of 2.6, 3.5, and 3.5 cm, respectively (Figure 4 and Figure S2). 

In this study, the inhibition percentages of the leaves and fruits methanolic extracts of *Z. spina-christi* L. for the three tested strains were calculated at C2 50 mg/mL, C3 100 mg/mL, C4 150 mg/mL, and C5 200 mg/mL after 10 days of incubation. The inhibition percentage for *A. alternata* ranged between 37.04 and 69.96% for leaves extract and 42.59 and 71.48% for fruits extract. For *A. citri*, the inhibition percentage ranged between 14.78 and 62.22% for leaves extract and 40.74 and 61.11% for fruits extract. The inhibition percentages for *A. radicina* were 43.78 to 69.48% for leaves extract (Table 4) and 19.68 to 57.43% for fruits extract. In general, leaves methanolic extracts were found to be the most potent form of in vitro antifungal activity.

### 3.4. Minimal Inhibitory Concentration (MIC)

The MIC of leaves and fruits methanolic extracts of *Z. spina-christi* L. against *A. alternata*, *A. citri*, and *A. radicina* strains was C5 200 mg/mL (Table 4).

### 3.5. Fungal Pathogenicity and Aggressiveness on Tomato Fruits

Table 5 shows the effect of methanolic extracts of *Z. spina-christi* leaves and fruits on the pathogenicity and aggressiveness of *A. alternata*, *A. citri*, and *A. radicina* strains on tomato fruits. Pathogenicity was increased when the skin was injured, as expected. In comparison to the controls, both extracts reduced the percentage of infected fruits. In the solvent control, methanol had no impact on fungal pathogenicity. With *A. alternata* and *A. citri*, the leaves extract was the most efficient, whether applied to wounded or unwounded fruits. When inoculated to tomato fruits, it prevented the growth of *A. alternata* and *A. citri* (pathogenicity). Only when applied to wounded or unwounded tomato fruits could the fruit extracts suppress the growth of *A. radicina*. In both wounded and unwounded fruits, the type of lesion was distinct. When a tomato fruit was wounded, the lesion was always the same size as the fungal colony’s diameter. A soft lesion outside the colony’s margin was noticed in the condition of inoculation without a wound. In both cases, the aggressiveness value obtained matched the entire lesion. Both extracts decreased the size of the *Alternaria* spp. lesion on tomato fruits, which was used to determine aggressiveness. On both wounded and unwounded fruits, the total suppression of lesions was measured in leaves extracts with *A. alternata* and *A. citri* but only on unwounded fruits with *A. radicina*. On the other hand, as compared to control, fruits extract fully inhibited lesions caused by *A. radicina* and reduced the lesions’ diameters caused by *A. alternata* and *A. citri* on wounded (0.5 and 0.4 cm) and unwounded (0.3 and 0.2 cm) tomato fruits.

## 4. Discussion

As described in the Section 3, there were 13 and 14 compounds with different retention times obtained from the HPLC chromatogram of methanolic extract of leaves and fruits, respectively. Polyphenolic compounds can be defined chemically as a substance that owns a benzene ring with one or more hydroxyl groups and is synthesized from the amino acid, phenylalanine, through the shikimic acid and phenylpropanoid pathways, where increased hydroxylation led to increased toxicity [20,21]. Likewise, polyphenols have a chief role in infighting microorganisms, herbivores, and insects, and phenolic compounds of most plant extracts act as active antifungal compounds [22]. We suggested that the higher content of gallic acid and ellagic acid in the leaves extract might have a key role as defensive phytochemicals with antifungal properties. It was reported that the presence of tannins (gallic acid, coffeic acid, ellagic acid, and catechin) in the leaves, bark, inner bark, fruits, and seeds of *Z. joazeiro* extract sample have pharmacological properties, while the flavonoids (quercetin and rutin) were described as having antioxidant properties [23]. Our findings were also in agreement with the findings of [24], who studied the damaging effects of *Alternaria solani* on the leaf surface of tomato plants and the role of gallic acid and its derivatives to alleviate this disease symptom. However, the predominant content of quercetin in the fruits extract could have anti-oxidative properties against fungal growth. In addition, quercetin had been documented to have the ability to antifungal activities against *Candida albicans* and *Aspergillus niger* [25]. Furthermore, quercetin might have a protective role by scavenging free radicals and enhancing the levels of antioxidants. In addition, the other lower-concentration polyphenolic compounds such as chlorogenic acid, methyl gallate, syringic acid, coumaric acid, vanillin, ferulic acid, naringenin, and cinnamic acid were found to have antimicrobial effects, which have definite modes of action, such as cell wall damage [26].

The data of GC-MS spectroscopy of methanol leaves extract revealed the presence of 13 components and the presence of 28 components for methanol fruits extract. The presence of Phenol, 2, 5-bis(1,1-dimethylethyl) is known to have antimicrobial activities [27,28]. Furthermore, it was reported to have antioxidant activity, anticancer, anti-inflammatory, antibacterial, and antiviral activity [29,30]. These combinations of the active components in the leaves methanolic extracts of *Z. spina-christi* were found to be biologically active and known to possess antibacterial, antifungal, and antioxidant properties. The presence of Decane, 2-methyl-(18.53%) has an antimicrobial effect according to [31]. Disulfide, di-tert-dodecyl (14.61%) was found to have antimicrobial activity [32]. Nonadecane (7.6%) has antioxidant, antibacterial, antimicrobial, antitoxic effects and is antimalarial, [33]. Aspidospermidin-17-ol, 1-acetyl-16-methoxy-(3.1%) had antimicrobial activity according to [34]. 1-Dodecene (2.87%) has antibacterial activity [35]. 2,6-Di-t-butyl-4-methylene-2,5-cyclohexadiene-1-one (2.21%) had antioxidant active compounds [36].

These bioactive compounds in fruits methanolic extract possess effective antifungal [37] and antimicrobial activity [38], including polysaccharides, which act as an osmoprotective mechanism of plants involved in immunity [39]; organic acids used as herbicides, utilized as a precursor for the synthesis of the antiviral drugs and antibacterial agents [40,41]; fatty acids which play an important role in fungal resistance [42,43]; and antioxidant components, which help the plant to protect itself against various types of oxidative damage [44].

The great efficacy of *Z. spina-christi* leaves and fruits methanol extracts against the pathogenic fungi *A. alternata*, *A. citri*, and *A. radicina* was due to the active phytochemical components. Our findings supported those of [45]: They examined antioxidant and antifungal activities of *Zizyphus* species leaves and discovered that methanolic leaf extracts had antifungal activity against (*Aspergillus flavus*, *A. niger*, and *A. alternata*). While these findings contradicted those of [46], the results of that investigation on the antifungal activity of ethanolic extracts of leaves from two *Ziziphus* species against *A. niger* revealed that the extracts did not affect the fungus. This could be because the antifungal activity of phytochemical components in plant extract differs according to the type of solvents used in extraction.

Several investigations have confirmed the antifungal activity of *Ziziphus* sp. leaves and fruits and other plant extracts against various pathogenic fungi. In agreement with our findings, the phytochemical screening of leaves from *Z. spina-christi* L. revealed tannins and flavonoid compounds that are physiologically active against the two root rot pathogens, *Drechslera biseptata* and *Fusarium solani*, in vitro [47]. Another study [48] found that methanolic leaves extracts of *Thymus vulgaris* and *Zingiber officinale* were highly active and had fungistatic and fungicidal effects on tomato phytopathogenic fungi *Fusarium oxysporum*, *Pythium aphanidermatum*, and *Rhizoctonia solani*, which are the causative agents of tomato damping-off diseases. They confirmed that carbendazim fungicide was more efficient than the two methanolic plant extracts at inhibiting the mycelial growth of all phytopathogenic fungi at 8 ppm, which was similar to our results. In addition, the findings of [49] are consistent with our findings. They discovered that *Ziziphus* genus leaves and fruit extracts are a rich source of natural bioactive compounds that could be useful in industrial and food applications. However, as compared to fruits extract, leaves extract exhibited the strongest antimicrobial activity and the highest level of total phenolic, flavonoids, and tannins. All bioactive compounds present in plant extracts act synergistically on fungi either by the inactivation of enzymes production, inhibition, or decrease in ergosterol content in filamentous fungi to enhance the overall antifungal activity [50,51].

The inhibition percentage for *A. alternata* was the highest with fruits extract (71.48%). For *A. citri*, the inhibition percentage reached 62.22% for leaves extract. The inhibition percentage was the highest (69.48%) for *A. radicina* for leaves extract. These findings agree with previous studies which reported that *Ziziphus* sp. leaves extract showed the highest total polyphenols content using different solvents of extraction compared to other plant parts [52,53,54], as well as to other plant species [55,56,57]. Phenolic compounds are typically found in the vacuoles of colorful tissues such as leaves and/or flower petals at the plant cell level [58]. Furthermore, earlier research has found that the lipophilic flavonoids found in the epidermis and/or cuticle of leaves are among the most efficient phenolic compounds providing plant resistance to a wide spectrum of phytopathogenic fungi [59]. According to [51], they found that the mycelial inhibition percentages of 5 *Solanum* species extracts against phytopathogenic *Curvularia lunata* were 64.8, 50.5, and 69.8% for fruit extract, leaf extract, and Captan fungicide, respectively.

The MIC values in earlier investigations varied depending on the fungus and plant species. The findings of [60,61] suggest that MICs of methanolic extracts of *Ziziphus mauritiana* Lam leaves were 125 g/mL and 500 g/mL for two strains of *Candida albicans*. The results of [51] showed that methanol extracts from the roots, stems, leaves, and fruits of *Solanum* species had antifungal effects against phytopathogenic *Curvularia lunata*. The MIC was 28.4 μg/mL. Various concentrations of the methanolic leaf extracts of *Z. spina-christi* L. were tested for antifungal activity against a variety of fungal strains [62]. The (MIC) for *Aspergillus niger* ATCC 102, *Aspergillus flavus* ATCC 247, and *Fusarium moniliform* ATCC 206 was 400mg/100mL [62]. The results reported by Alotibi [13] confirmed that a 50 mg/mL concentration of *Z. spina-christi* aqueous extract strongly reduced the development of *Helminthosporium rostratum*, *Alternaria brassicae*, and *Rhizoctonia solani*.

Similar to our results, previous reports confirmed the efficacy of different plant extracts against the pathogenicity of different fungi because secondary plant metabolites have a marked potential as a resource of effective antifungal agents [63,64,65]. Spraying rice plants with *Nerium oleander* leaf extract considerably reduced the occurrence of brown spots (caused by *Bipolaris oryzae*) by 52%, according to [66]. Our findings are in harmony with those of [67], who found that an extract of *Aeglemarmelos* reduced fruit spot disease in *Lycopersicum esculentum* by 61.29%. The antifungal activity of ethanolic and chloroformic extracts of *Eucalyptus globulus* and *Calendula officinalis* was confirmed on the development of *Alternaria alternata* and *A. arborescens*, as well as the pathogenicity of tomato fruits [19].

## 5. Conclusions

In general, the leaves methanolic extract was found to be the most potent form of in vitro antifungal activity and control of pathogenicity and aggressiveness of *A. alternata*, *A. citri*, and *A. radicina* strains on tomato fruits. These results could be attributed to the presence of bioactive and polyphenol compounds, which can provide promising information for the potential use of their extract in the treatment of the three tested *Alternaria* spp. infections. It was the first time the biocontrol efficacy the methanolic extracts of *Z. spina-christi* leaves and fruits was recorded against the in vitro growth and pathogenicity of *A. alternata*, *A. citri*, and *A. radicina* on tomato fruits. The outputs of this study suggest the potential use of *Z. spina-christi* leaves’ and fruits’ methanolic extracts as antioxidants in pharmaceutical manufacture. The antifungal properties of the extracts also validate their potential applications as fungistatic and fungicide on tomato fruits.

## Figures and Tables

**Table 1:** Polyphenols compounds determined by HPLC in crude methanolic extracts of *Z. spina-christi* leaves and fruits.

Compound	Leaves Extracts			Fruits Extracts		
	RT	Relative Area (%)	Conc. (µg/mL)	RT	Relative Area (%)	Conc. (µg/mL)
Gallic acid	3.058	25.62 ± 0.12 **	275.55 ± 0.65 **	3.036	19.73 ± 0.16 **	7.73 ± 0.02 **
Chlorogenic acid	3.824	1.06 ± 0.006 **	8.79 ± 0.01 **	3.927	3.51 ± 0.02 **	1.06 ± 0.01 **
Catechin	3.974	2.32 ± 0.006 **	25.99 ± 0.01 **	4.092	2.13 ± 0.03 **	0.87 ± 0.01 **
Methyl gallate	4.781	1.00 ± 0.015 **	1.40 ± 0.01 **	5.202	4.69 ± 0.04 **	0.24 ± 0.01 **
Coffeic acid	5.294	8.17 ± 0.03 **	32.38 ± 0.32 **	5.622	1.92 ± 0.04 **	0.28 ± 0.01 **
Syringic acid	5.857	1.49 ± 0.006 **	7.64 ± 0.05 **	5.905	2.91 ± 0.04 **	0.54 ± 0.01 **
Pyro catechol	6.068	ND	ND	6.068	ND	ND
Rutin	7.131	15.23 ± 0.12 **	199.84 ± 0.16 **	7.157	2.96 ± 0.04 **	1.42 ± 0.03 **
Ellagic acid	7.822	25.56 ± 0.15 **	350.87 ± 0.33 **	7.831	8.89 ± 0.11 **	4.45 ± 0.13 **
Coumaric acid	8.389	3.12 ± 0.02 **	4.57 ± 0.16 **	8.132	2.16 ± 0.02 **	0.12 ± 0.01 **
Vanillin	8.789	1.05 ± 0.02 **	2.40 ± 0.02 **	9.147	0.95 ± 0.03 **	0.08 ± 0.002 **
Ferulic acid	9.556	4.86 ± 0.05 **	20.25 ± 0.05 **	9.812	1.96 ± 0.01 **	0.30 ± 0.02 **
Naringenin	10.094	9.77 ± 0.23 **	52.61 ± 0.28 **	10.126	1.13 ± 0.03 **	0.22 ± 0.015 **
Querectin	12.995	0.75 ± 0.03 **	5.61 ± 0.19 **	13.128	44.21 ± 0.30 **	12.00 ± 0.11 **
Cinnamic acid	14.303	ND	ND	14.202	2.84 ± 0.08 **	0.10 ± 0.01 **
Kaempferol	15.495	ND	ND	15.495	ND	ND
Hesperetin	16.075	ND	ND	16.075	ND	ND

ND = Not Detected; Values are means of three replicates ± standard deviation; Statistical significance of differences compared to control: ** Significant at *p* < 0.01 according to Duncan test.

**Table 2:** Phytochemical components in methanolic leaves extract of *Z. spina-christi* identified by GC-MS spectroscopy.

No.	Band/RT	Compound Name	Molecular Formula	Area Percent % *
1	20.903	2,6-Di-t-butyl-4-methylene-2,5-cyclohexadiene-1-one	C_15_H_22_O	2.21 ± 0.006
2	21.098	Decane, 2-methyl-	C_11_H_24_	18.53 ± 0.29
3	21.326	Cycloheptasiloxane, tetradecamethyl-	C_14_H_42_O_7_Si_7_	1.1 ± 0.07
4	23.438	18-Methyl-nonadecanol, trimethylsilyl ether	C_23_H_50_OSi	1.27 ± 0.03
5	23.838	Nonadecane	C_19_H_40_	7.6 ± 0.15
6	24.474	Dodecane, 1-fluoro-	C_12_H_25_F	2.36 ± 0.04
7	26.087	Disulfide, di-tert-dodecyl	C_24_H_50_S_2_	14.61 ± 0.1
8	26.717	9,12,15-Octadecatrienoic acid, 2-[(trimethylsilyl)oxy]-1-[[(trimethylsilyl)oxy]m	C_27_H_52_O_4_Si_2_	0.58 ± 0.02
9	28.582	1-Dodecene	C_12_H_24_	2.87 ± 0.075
10	30.247	Aspidospermidin-17-ol, 1-acetyl-16-methoxy-	C_22_H_30_N_2_O_3_	3.1 ± 0.15
11	32.736	1-Monolinoleoylglycerol trimethylsilyl ether	C_27_H_54_O_4_Si_2_	1.19 ± 0.03
12	33.509	6-epi-shyobunol	C_15_H_26_O	1.84 ± 0.07
13	37.331	Phenol, 2,5-bis(1,1-dimethylethyl)-	C_14_H_22_O	40.24 ± 0.37

* Values are means of three replicates ± standard deviation.

**Table 3:** Phytochemical components in methanolic fruits extract of *Z. spina-christi* identified by GC-MS spectroscopy.

No.	Band/RT	Compound Name	Molecular Formula	Area Percent %
1	18.248	Furan-2-carboxylic acid, 3-methyl-, trimethylsilyl ester	C_9_H_14_O_3_Si	2.27 ± 0.03
2	18.397	5-Fluoroveratraldehyde	C_9_H_9_FO_3_	0.56 ± 0.02
3	18.557	2-Trimethylsiloxy-6-hexadecenoic acid, methyl ester	C_20_H_40_O_3_Si	0.25 ± 0.02
4	23.055	Melezitose	C_18_H_32_O_16_	0.26 ± 0.01
5	23.444	Cyclooctasiloxane, hexadecamethyl-	C_16_H_48_O_8_Si_8_	0.94 ± 0.04
6	24.039	2,2-Dimethyl-5-[2-(2-trimethylsilylethoxymethoxy)-propyl]-[1,3]dioxolane-4-carbo	C_15_H_30_O_5_Si	8.46 ± 0.10
7	24.84	D-Erythro-Pentofuranose, 2-deoxy-1,3,5-tris-O-(trimethylsilyl)-	C_14_H_34_O_4_Si_3_	15 ± 0.20
8	25.189	D-Erythrotetrofuranose, tris-O-(trimethylsilyl)-	C_13_H_32_O_4_Si_3_	2.48 ± 0.11
9	25.338	D-mannonic acid, 2,3,5,6-tetrakis-o-(trimethylsilyl)-, γ-lactone	C_18_H_42_O_6_Si_4_	22.72 ± 0.2
10	25.498	Mannofuranoside, methyl 2,3,5,6-tetrakis-O-(trimethylsilyl)-, α-D-	C_19_H_46_O_6_Si_4_	4.6 ± 0.10
11	25.664	Methyl α-D-glucofuranoside, 4TMS derivative	C_19_H_46_O_6_Si_4_	11.18 ± 0.2
12	26.03	{2,2-Dimethyl-5-[2-(2-trimethylsilylethoxymethoxy)propyl][1,3]dioxolan-4-yl}meth	C_15_H_32_O_5_Si	0.46 ± 0.02
13	26.179	Lyxose, tetra-(trimethylsilyl)-ether	C_17_H_42_O_5_Si_4_	2.04 ± 0.04
14	26.265	Glucopyranose, 1,2,3,4,6-pentakis-O-(trimethylsilyl)-, D-	C_21_H_52_O_6_Si_5_	3.62 ± 0.22
15	26.722	9,12,15-Octadecatrienoic acid, 2-[(trimethylsilyl)oxy]-1-[[(trimethylsilyl)oxy]m	C_27_H_52_O_4_Si_2_	0.66 ± 0.05
16	26.78	7,9-Diethyl-2,4-bis(dimethylamino)-10-imino-8-thio-1,7,9-triazaspiro[4.5]-1,3-de	C_15_H_24_N_6_OS	1.25 ± 0.14
17	27.094	beta.-D-(+)-Talopyranose, 5TMS derivative	C_21_H_52_O_6_Si_5_	0.49 ± 0.05
18	30.098	cis-13-Octadecenoic acid	C_18_H_34_O_2_	2.01 ± 0.02
19	31.695	Octasiloxane, 1,1,3,3,5,5,7,7,9,9,11,11,13,13,15,15-hexadecamethyl-	C_16_H_50_O_7_Si_8_	0.94 ± 0.03
20	32.193	5,8,11-Eicosatrienoic acid, (Z)-, TMS derivative	C_23_H_42_O_2_Si	0.31 ± 0.05
21	32.37	Heptanedioic acid, bis(trimethylsilyl) ester	C_13_H_28_O_4_Si_2_	2.35 ± 0.06
22	32.462	D-(+)-Turanose, octakis(trimethylsilyl) ether	C_36_H_86_O_11_Si_8_	6.52 ± 0.29
23	32.593	Arabino-hexonic acid, 2-deoxy-3,5,6-tris-o-(trimethylsilyl)-, γ-lactone	C_15_H_34_O_5_Si_3_	1.02 ± 0.02
24	32.748	Aucubin, hexakis(trimethylsilyl) ether	C_33_H_70_O_9_Si_6_	1.76 ± 0.19
25	33.223	9,12,15-Octadecatrienoic acid, 2-[(trimethylsilyl)oxy]-1-[(trimethylsilyl)oxy]m	C_27_H_52_O_4_Si_2_	1.27 ± 0.11
26	33.72	SILIKONFETT	N.I.	2.02 ± 0.03
27	36.313	1-Monolinoleoylglycerol trimethylsilyl ether	C_27_H_54_O_4_Si_2_	0.29 ± 0.02
28	42.103	Heptasiloxane, 1,1,3,3,5,5,7,7,9,9,11,11,13,13-tetradecamethyl-	C_14_H_44_O_6_Si_7_	0.29 ± 0.01

N.I. Not Identified. Values are means of three replicates ± standard deviation.

**Table 4:** The inhibition percentage of leaves and fruits extracts of *Zizyphus spina-christi* L. against *A. alternata*, *A. citri* and *A. radicina.*

Fungi	Concentration (mg/mL)	Leaves Extract	Fruits Extract	Mancozeb
*A. alternata*	50	37.04	42.59	
	100	42.59	59.26	97.8
	150	44.44	50.00	
	200	62.96	71.48	
*A. citri*	50	14.78	40.74	
	100	21.44	37.04	94.4
	150	33.33	40.74	
	200	62.22	61.11	
*A. radicina*	50	43.78	19.68	
	100	53.82	23.69	83.13
	150	57.83	47.79	
	200	69.48	57.43	

**Table 5:** Effect of *Z. spina-christi* L.leaves and fruits extracts on pathogenicity and aggressiveness of *Alternaria alternata*, *A. citri*, and *A.radicina* on tomato fruits.

Treatments	*A. alternata*				*A. citri*				*A. radicina*			
	Pathogenecity ^1^		Aggressiveness ^2^		Pathogenecity ^1^		Aggressiveness ^2^		Pathogenecity ^1^		Aggressiveness ^2^	
	W	U	W	U	W	U	W	U	W	U	W	U
Positive control ^3^	100	70	1.5	1.2	100	64	1.8	1.4	100	58	1.9	1.7
Negative control ^4^	0	0	0	0	0	0	0	0	0	0	0	0
Solvent control ^5^	100	70	1.5	1.1	100	60	1.9	1.5	100	59	2	2.1
Leaves extract	0	0	0	0	0	0	0	0	10	0	1.2	0
Fruits extract	30	11	0.5	0.3	20	15	0.4	0.2	0	0	0	0

^1^ % of infected fruits. Mean of two independent experiments. ^2^ Mean lesion diameter (cm). Mean of 10 replicates from two independent experiments. ^3^ Inoculated fruits untreated with plant extract or ethanol. ^4^ Uninoculated fruits sprayed with ethanol. ^5^ Inoculated fruits sprayed with ethanol. W—wounded; U—unwounded.

## Data Availability

The datasets generated and/or analyzed during the current study are available from the corresponding author upon reasonable request.

## Supplementary Materials

The following supporting information can be downloaded at: https://www.mdpi.com/article/10.3390/plants11060746/s1, Figure S1: A. Leaves methanolic extract, B. Fruits methanolic extract of *Ziziphus spina-christi* L and C. Graphical abstract of the study; Figure S2: In vitro antifungal activities of methanol leaves and fruits extracts of *Zizyphus spina-christi* L. against A. *Alternaria alternata* and fruits extract and B. *A. radicina* and leaves extracts after 10 days of incubation at 28 °C. Plates of control (fungus only) and positive control (Mancozeb 3%) are also shown; Table S1: Concentrations of polyphenols standard estimated by HPLC.
