# Supplementary material for: Bioactive Compounds and Antifungal Activity of Leaves and Fruits Methanolic Extracts of Ziziphus spina-christi L
Source: Plants (Basel). 2022 Mar 11;11(6):746. doi: 10.3390/plants11060746 (PMC8955299; doi:10.3390/plants11060746)
Supplement: Supplementary file 1 [file plants-11-00746-s001.zip › plants-1555678-supplementary.pdf]

Supplementary Materials:

Figure S1. A. Leaves methanolic extract, **B**. Fruits methanolic extract of *Ziziphus spina-christi* L and **C**. Graphical abstract of the study.

A. Leaves methanolic extract

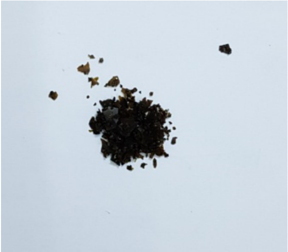

B. Fruits methanolic extract

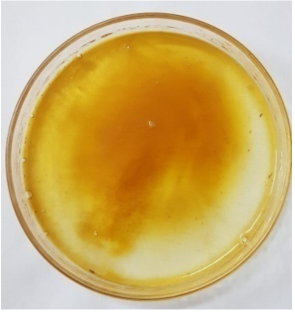

C.

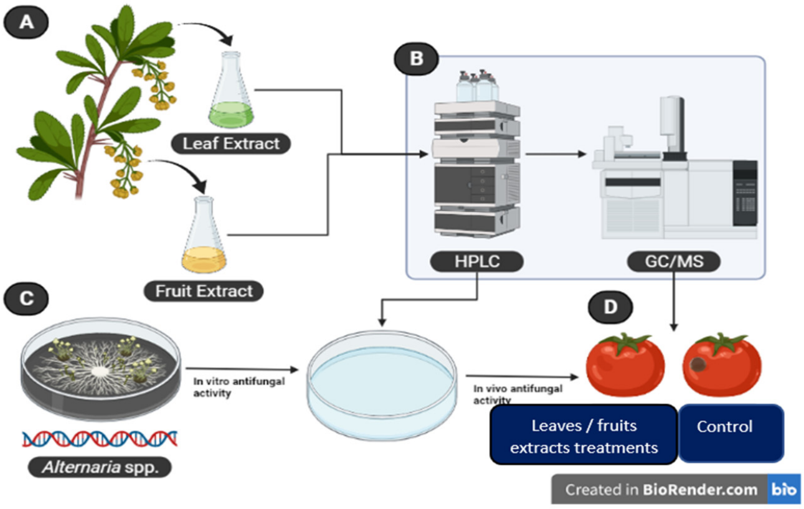

Figure S2. In vitro antifungal activities of methanol leaves and fruits extracts of *Zizyphus spina-christi* L. against (A) *A. alternata* and fruits extract and (B) *A. radicina* and leaves extracts after 10 days of incubation at 28°C. Plates of control (fungus only) and positive control (Mancozeb 3%) are also shown.

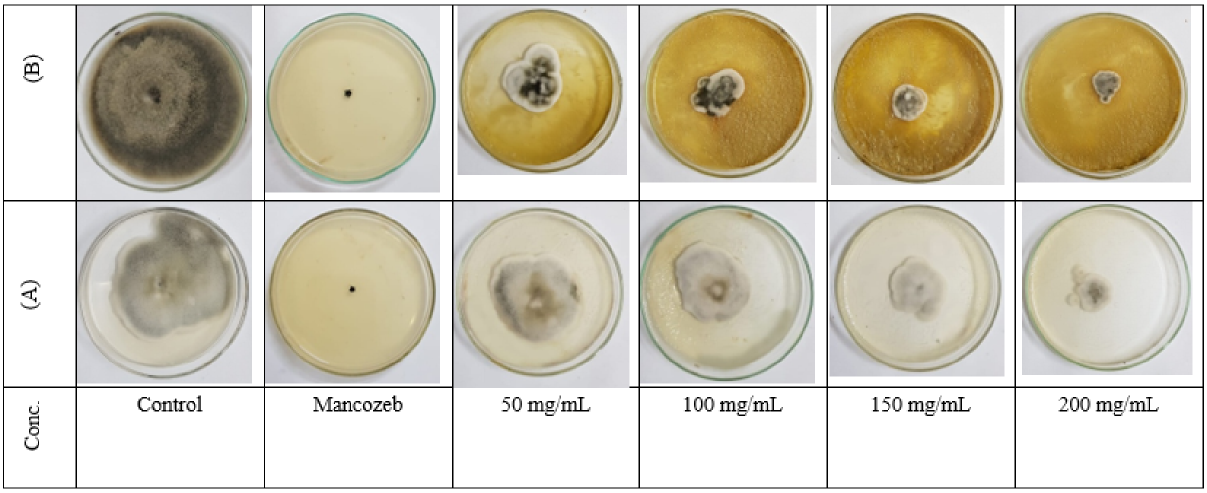

Table S1. Concentrations of polyphenols standard estimated by HPLC.

	Standard	
	Conc. (µg/ml)	Area
Gallic acid	16.8	80.43
Chlorogenic acid	28	173.11
Catechin	67.5	310.33
Methyl gallate	10.2	373.38
Coffeic acid	18	233.72
Syringic acid	17.2	172.47
Pyro catechol	29.2	217.76
Rutin	61	239.43
Ellagic acid	34.3	128.65
Coumaric acid	13.2	464.04
Vanillin	12.9	289.87
Ferulic acid	12.4	153.36
Naringenin	15	143.44
Querectin	12.8	88.51
Cinnamic acid	5.8	298.17
Kaempferol	12	108.13
Hesperetin	13.2	204.91
